# Performance of a multianalyte test as an aid for the diagnosis of ovarian cancer in symptomatic women

**DOI:** 10.1186/1479-5876-10-45

**Published:** 2012-03-12

**Authors:** Dominic J Autelitano, Linda Raineri, Kate Knight, Kelly Bannister, Gregory E Rice

**Affiliations:** 1 Healthlinx Ltd, 576 Swan St., Richmond, VIC 3121, Australia; 2 University of Queensland Centre for Clinical Research, Royal Brisbane and Women's Hospital Campus, Herston, QLD 4029, Australia

**Keywords:** Ovarian cancer, Tumour markers, Multianalyte, Diagnostic

## Abstract

**Background:**

Concomitant with the development of *in vitro *diagnostic multivariate index assays (IVDMIAs) to improve the diagnostic efficiency of ovarian cancer detection is the need to identify appropriate biostatistical approaches to assess improvements in risk predication. In this study, we assessed the utility of three different approaches for comparing diagnostic efficiency of an ovarian cancer multivariate assay in a retrospective case - control phase 2 biomarker trial. The control cohort included both disease-free women and women with benign gynecological conditions to more accurately reflect the target population of symptomatic women.

**Methods:**

The study cohort comprised plasma samples from 244 healthy controls, 223 women with benign gynecological conditions, 53 borderline ovarian cancer cases and 222 women with malignant epithelial ovarian cancer. A multivariate classification model was developed that incorporated plasma concentrations of CA125, C-reactive protein (CRP), serum amyloid-A (SAA), interleukin-6 (IL6) and interleukin-8 (IL8) that were measured using *in vitro *diagnostics assays on medical device approved clinical analysers. The posterior probability values derived from the implemented algorithm were used for comparisons of the diagnostic performance between the multianalyte panel and CA125 using multiple methods; area under the curve (AUC) of the receiver operating characteristics curve, integrated discrimination improvement (IDI) and net reclassification improvement (NRI).

**Results:**

Each of the biomarkers displayed significantly elevated plasma concentrations in malignant ovarian cancer patients compared with either benign or control subjects. For the discrimination of borderline and malignant ovarian cancer from control and benign subjects, the multivariate classification model showed a significantly greater AUC than that for CA125 alone (88.4% versus 84.3%, respectively, *p *< 0.001). At a posterior probability threshold of 0.5, the IVDMIA delivered a specificity of 92.3% and a sensitivity of 76.4%. When set at a specificity of 95%, the multimarker diagnostic delivered a sensitivity of 69.5% compared with 62.5% for CA125. Enhanced diagnostic performance of the IVDMIA over the use of CA125 alone was confirmed statistically by alternative comparisons using IDI and NRI.

**Conclusions:**

This study confirms in an independent sample set that a blood-based multianalyte assay has significant advantages over CA125 for distinguishing symptomatic women with borderline and malignant ovarian cancer from controls or those with benign disease.

## Background

Estimates by the International Agency for Research on Cancer indicate that the number of new cases of ovarian cancer for the year 2008 would reach 225,000 with 140,000 expected deaths from this disease in the same period [1]. In the USA, it is estimated that ovarian cancer will account for 13,850 deaths in 2010, making it the fifth most lethal malignancy in females [2]. Of particular significance is the fact that the distribution of ovarian cancers by stage at the time of diagnosis is dramatically skewed towards late stage disease, with only approximately 30% of ovarian cancers diagnosed when tumours represent localised or regionally contained disease [2]. Overall, five-year survival rate for patients in the USA diagnosed across all stages of ovarian cancer is 46% [2]. Five-year survival rates among patients diagnosed with localised disease, however, are around 94%, but fall to 73% in patients diagnosed with regional malignancy and are only 28% in patients diagnosed with late stage disease [2]. These data are consistent with the proposal that patients diagnosed with early stage ovarian malignancies have a distinct survival advantage and raise the possibility that improved methods to detect more early stage ovarian malignancies may provide improved clinical outcomes.

Due to low incidence of ovarian cancer in most developed populations (1 per 2,500 women per year), it has been suggested that for an acceptable ovarian cancer screening test to be implemented, it would need to perform with a minimum specificity of 99.6% to achieve a positive predictive value of 10% for screening the general population of post-menopausal women [3]. At present, no screening method has been demonstrated to be sufficiently robust to allow for population based screening for ovarian cancer.

CA125, a high molecular weight glycoprotein remains the most widely used biomarker for confirmation of diagnosis and management of ovarian cancer. Although it is commonly used as an aid in the diagnosis of ovarian malignancy, it has significant limitations in terms of sensitivity and specificity. A review of pre-operative serum CA125 concentrations in ovarian cancer patients by FIGO stage and by histological type showed that CA125 was elevated in only 50% of stage I ovarian cancer cases and in 69% of mucinous ovarian tumours while being far more prominently expressed in patients with late stage serous tumours [4]. Elevation of circulating CA125 concentrations have also been documented in benign gynecological conditions, pregnancy and other malignancies, making CA125 less useful as a selective biomarker for the detection of ovarian cancer [4].

Some improvement in the preoperative diagnosis of ovarian cancer has been achieved by combining serum CA125 concentration, ultrasound score and menopausal status into a risk of malignancy index (RMI) which was shown to outperform the use of CA125 alone to discriminate between a benign and malignant pelvic mass [5]. Furthermore, since as many as 20% of ovarian cancers express little or no CA125, it is likely that additional secreted biomarkers may be able to complement the use of CA125 to improve diagnostic efficiency [6]. Different approaches have been taken to test the use of various multimarker panels that include CA125 to generate a multivariate model to predict the likelihood of ovarian cancer in various patient cohorts. Combinations of biomarkers and multivariate analyses have demonstrated increases in diagnostic efficiency for predicting ovarian malignancy in comparison to using CA125 alone [7-13]. Several of these multimarker tests are aimed at more accurately distinguishing between malignant and benign adnexal masses, thus allowing for more streamlined triage of these patients [10,11,14].

Previously, we reported the results of a retrospective case-control study that assessed the performance of a five biomarker panel (CA125, CRP, SAA, IL6 and IL8) and demonstrated increased diagnostic efficiency of this panel over CA125 alone as assessed by the area under the receiver operating characteristic curve (AUC) using a bootstrapping procedure [8]. This initial multianalyte approach was limited to a comparison of normal healthy controls with confirmed cases of malignant ovarian cancer and demonstrated significant diagnostic advantage over CA125 for detection of both early and late stage ovarian malignancy.

A recent trend in the development of more efficient diagnostic tests has been the use of algorithm-base multivariate index assays. With the development of this new class of diagnostic, the discipline has sought new biostatistical approaches for assessing and quantifying incremental gains in diagnostic efficiency. Traditionally, the AUC has been used as a measure and comparator of diagnostic efficiency. Several investigators have argued that this measure alone may be imperfect and inefficient for comparing the true clinical usefulness of alternative marker panels [15,16]. It was observed that when evaluating improvement in risk assignment of biomarkers, very large odds ratios were often associated with very small increases in the AUC. This feature of the receiver operator characteristic curve analysis limits its utility in identifying putative beneficial contributions of new biomarkers to algorithm-based models. As a result, alternative methods for comparison of diagnostic efficiency have been developed and successfully applied including integrated discrimination improvement (IDI) that assesses improvement in risk discrimination based on the integral of sensitivity and specificity of all possible thresholds [15,16]. These methods were initially developed for the derivation of prognostic indicators from prospective cohort studies, but have been applied in the context of developing diagnostic indicators from case-control studies [17-19].

To further validate the efficacy of the previously described multianalyte panel and to test the utility of different approaches for assessing diagnostic efficiency, we have evaluated the performance of the IVDMIA on an independent cohort of 742 patient samples that included a significant proportion of benign gynecological pathologies that more accurately defines the target population. The performance of the IVDMIA was compared to CA125 alone using AUC bootstrapping approaches as well as IDI and NRI in order to determine the benefit of the multimarker model in correctly classifying women who present clinically with symptoms of ovarian cancer.

## Methods

### Study samples

The study population comprised 244 apparently healthy normal women, 223 patients with benign gynecological conditions, 53 patients with borderline ovarian tumours and 222 patients with malignant ovarian tumours (Table 1).

**Table 1:** Characteristics of the study population

	No. of samples	Stage					Age (years)		
		**I**	**II**	**III**	**IV**	**UNK**	**Range**	**Median**	**Mean (SD)**

**Normal**	244						19-85	50	51 (13)

**Benign**	223						20-90	47	49 (15)

**Borderline**	53						19-79	47	48 (14)

**Malignant**	222						22-88	58	58 (14)

Ser	130	14	14	80	12	10			

Endo	19	7	6	4	-	2			

Muc	16	8	0	3	-	5			

CC	16	8	1	7	-	-			

Other	41	5	6	12	2	16			

Staging data were unavailable for a proportion of the diagnosed malignant cases (UNK).

All patients underwent surgical removal of ovarian mass or cysts and pathology examination of tissue sections was used to provide a definitive diagnosis. Patients classified as malignant were women with histologically confirmed epithelial ovarian cancer patients and patients classified as benign consisted of women diagnosed with a range of common benign gynecological conditions including serous and mucinous cystadenoma, cystadenofibromas, endometriotic cysts, follicular cysts, fibrothecoma and endometriosis.

Plasma samples were collected with consent from healthy women or from patients prior to surgery or treatment and no special conditions were implemented in preparation for blood collection. The overall protocol was approved by the Mercy Hospital for Women Human Research and Ethics Committee (R09/06). The majority of the samples were provided from banked sample collections at the National University of Singapore, Victorian Cancer Biobank and the West Australian Research and Tissue Network from which part of the samples were collected via the Australian Ovarian Cancer Study. Samples obtained from normal apparently healthy women were collected with consent by ARL pathology as part of the study design. A further 234 samples were collected from gynecological oncology patients attending specialist clinics at the Mater Hospital, Brisbane, Australia and the Women's Clinic, Southend University Hospital, Essex, UK under local ethics approvals. Blood samples were collected into EDTA vacutainer tubes and samples stored as 250-1000 μL aliquots at -80°C until required for analysis. Samples were thawed once, dispensed into single use assay aliquots and were re-labelled to create a totally blind set for biomarker analysis. The order of assay for each blinded sample was further randomised to remove any possible assay bias.

### Study design

The present study was a case-control retrospective trial design to test the efficacy of a biomarker panel to detect ovarian cancer in symptomatic women, similar to that previously described [8]. The study cohort included both confirmed cases of malignant epithelial ovarian cancer, borderline ovarian tumours as well as control patients with benign gynecological pathologies. A group of apparently healthy age-matched normal women have been included as part of the control cohort.

### Biomarker quantitation

Five biomarkers, CA125, CRP, SAA, IL6 and IL8, previously associated with ovarian cancer [8] were analysed in each sample using the clinical pathology platforms Immulite and BN-II (Siemens Healthcare Diagnostics). CA125 (Siemens OM-MA assay), IL6 and IL8 were analysed on the Immulite while CRP and SAA were analysed on the BN-II platform. Analytes were analysed sequentially on each instrument from a single sample aliquot to avoid multiple freeze-thaw cycles and sample variation. All assays were performed as per the manufacturer's instructions. QC measurements were within the expected ranges and coefficients of variation for the assays performed were less than 5% (CA125, CRP, IL6 and IL8) and 8.2% (SAA). The limit of analytical sensitivity as specified by the manufacturer was 1 U/mL (CA125), 0.15 mg/L (CRP), 0.80 mg/L (SAA), 1 pg/mL (IL6) and 2.5 pg/mL (IL8). In the event that sample determinations delivered values at the limit of analytical sensitivity of the assay, values were entered for analysis as limit of sensitivity/2 so that a definitive lower value could be used in statistical analyses.

### Statistical analysis

Statistical comparison of multiple groups was assessed using the Kruskal-Wallis test and Dunn's multiple comparison was employed as a post-hoc test to determine differences between groups. For two sample group comparisons, statistical significance was determined using the Mann Whitney test (GraphPad Prism, La Jolla, CA, USA). Comparison of patient age across the groups was performed using one-way ANOVA followed by Tukey's multiple comparison test. In all cases, a *p *value < 0.05 was considered to be statistically significant.

### Multivariate modelling

Multivariate model development and statistical comparisons of biomarker models were performed by an independent biostatistician (Emphron Informatics Pty Ltd, Toowong, Qld, Australia). A multivariate classification model that incorporated all five biomarkers was developed using a stochastic gradient boosting model with a logistic loss function as previously described [8]. The implemented classification algorithm reported a posterior probability value (*i.e*. the likelihood that a sample came from a woman with ovarian cancer) for each patient sample using Leave-One-Out-Cross Validation [20]. The cross-validated predicted probabilities were used to generate the ROC curve for the IVDMIA. Comparisons between the diagnostic efficiency of CA125 alone and the IVDMIA were first tested by assessing AUC as calculated using the Wilcoxon statistic [21]. As the AUC for CA125 and for the biomarker panel are not statistically independent, since they are based on the same patients, the difference in AUC between the diagnostics were statistically assessed using a bootstrap procedure [22]. The number of bootstrap samples used in this analysis was n = 10,000, the estimators considered were the AUC as well as the difference between the AUC's, and the measures of accuracy were the 95% confidence intervals.

Statistical differences in diagnostic efficiency between CA125 and the IVDMIA were further assessed using: IDI that is based on a measure of separation in predicted probabilities for case and control groups; and NRI that assesses reclassification tables and quantifies the correct movement in categories [15,16]. Although originally developed in the context of assessing the contribution of additional markers to prognostic indicators using prospective cohort studies, these techniques have been adapted for retrospective case control studies [17-19].

## Results

### Characteristics of the study population

The age range for the normal control, benign, borderline and malignant ovarian cancer groups was similar (Table 1), however, the mean age of the malignant ovarian cancer group was significantly higher compared with the other groups (51 vs 49 vs 48 vs 58 respectively, *p *< 0.001). The control, non-malignant cohort was made up of 244 (52.2%) normal control women and 223 (47.8%) patients with benign gynecological lesions with a mean age of 50 ± 14 (SD). The malignant ovarian cancer cohort comprised 130 (58.5%) serous, 19 (8.6%) endometrioid, 16 (7.2%) mucinous, 16 (7.2%) clear cell and 41 (18.5%) of other types that included predominantly mixed forms and adenocarcinomas with no specific histotype recorded (Table 1). A total of 33 (14.9%) of malignant ovarian cancer samples had no staging data reported, 42 (18.9%) were diagnosed with Stage I disease, 27 (12.2%) with Stage II, 106 (47.7%) Stage III and 14 (6.3%) with Stage IV disease (Table 1).

### Plasma biomarker concentrations in control, benign, borderline and malignant ovarian cancer patients

**Figure 1:**
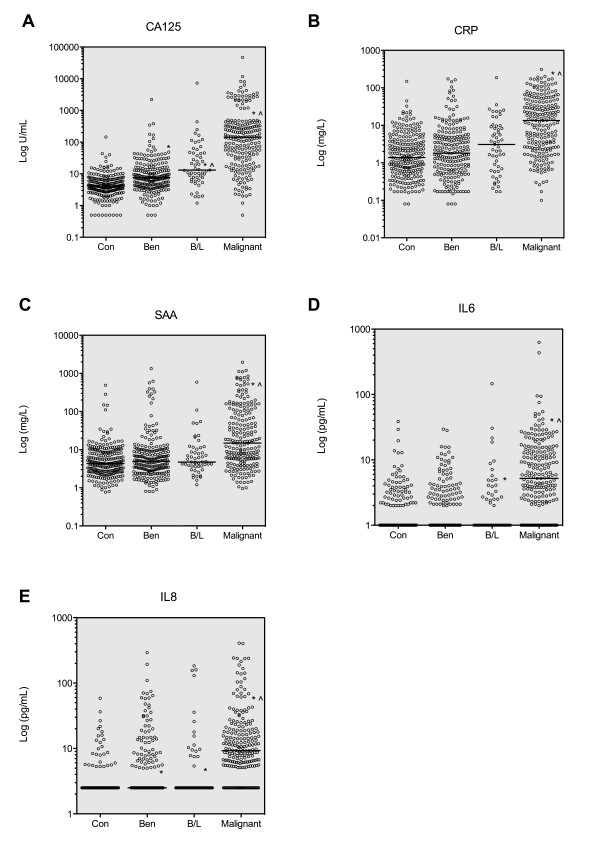
**Comparison of individual patient plasma biomarker concentrations across all patient groups**. A total of 244 normal controls (Con), 223 patients with benign gynecological conditions (Ben), 53 patients with borderline ovarian tumours (B/L) and 222 cases of malignant epithelial ovarian cancer (Malignant) were analysed. Open circles indicate the individual values of each patient measurement shown on a log scale and horizontal lines show median concentration of each group. *: *p *< 0.05, compared with Con group; ^: *p *< 0.05 compared with Ben group (Kruskal-Wallis test followed by Dunn's multiple comparison test)

The distribution of plasma concentrations of each tumour marker in individual patient samples is shown in Figure 1. Circulating concentrations of all measured biomarkers showed a significant difference (*p *< 0.05) between normal control samples and patients with malignant disease. Each biomarker also demonstrated a significant difference in plasma concentrations between benign and malignant tumour groups (*p *< 0.05), indicating that these analytes could discriminate between either normal controls or benign cases and those with malignant epithelial ovarian carcinoma. Concentrations of CRP, SAA and IL6 were indistinguishable between normal controls and patients with benign conditions. Patients with borderline ovarian tumours displayed significantly elevated concentrations of CA125 compared with either normal controls or benign patients, while IL6 and IL8 concentrations were slightly elevated in borderline patients compared with normal controls (Figure 1 and Table 2).

**Table 2:** Plasma biomarker concentrations

Biomarker		Normal Control (n = 244)	Benign (n = 223)	Borderline (n = 53)	Early Stage Malignant (Stages I & II) (n = 69)	Late Stage Malignant (Stages III & IV) (n = 120)
CA-125	Mean ± SE	6.05 ± 10.4	27.35 ± 10.2	180.3 ± 137	380.9 ± 97	1337.0 ± 420
	
(U/mL)	Median (range)	4.10 (0.5-146)^a^	7.60 (0.5-2219)^a^	13.1 (1.2-7266)^a, b^	52.3 (0.5-4277)^a, b^	208.5 (3.3-47206)^a, b^

CRP	Mean ± SE	3.99 ± 0.7	8.20 ± 1.5	10.05 ± 3.6	16.73 ± 3.4	39.65 ± 4.5
	
(mg/L)	Median (range)	1.38 (0.08-149.4)	1.81 (0.08-172.7)	3.09 (0.17-186.7)	4.77 (0.10-139.1)^a, b^	21.80 (0.30-309.5)^a, b^

SAA	Mean ± SE	10.82 ± 2.5	29.00 ± 7.9	22.11 ± 11.3	47.47 ± 13.3	127.7 ± 24.3
	
(mg/L)	Median (range)	4.34 (0.77-489.0)	4.95 (0.80-1337)	4.74 (1.20-593.2)	8.50 (1.00-737.0)^a, b^	24.23 (0.96-1958)^a, b^

IL-6	Mean ± SE	2.11 ± 0.23	2.61 ± 0.25	6.26 ± 2.79	8.54 ± 1.71	20.09 ± 6.29
	
(pg/ml)	Median (range)	1.00 (1.00-38.50)	1.00 (1.00-29.50)	1.00 (1.00-146.0)	4.10 (1.00-93.70)^a, b^	8.45 (1.00-628.0)^a, b^

IL-8	Mean ± SE	3.73 ± 0.33	10.27 ± 1.81	16.83 ± 5.67	19.27 ± 4.88	29.36 ± 5.88
	
(pg/mL)	Median (range)	2.50 (2.50-58.70)	2.50 (2.50-292.0)^a^	2.50 (2.50-183.0)^a^	2.50 (2.50-239.0)^a, b^	6.10 (2.50-408.0)^a, b^

Data are presented as mean ± SE and median (range). ^a^: p < 0.05 compared with normal controls and ^b^: p < 0.05 compared with benign group (Kruskal-Wallis followed by Dunn's Multiple Comparison test). Note that 33 malignant ovarian cancer samples were excluded from early/late stage analysis since stage data was not recorded.

**Figure 2:**
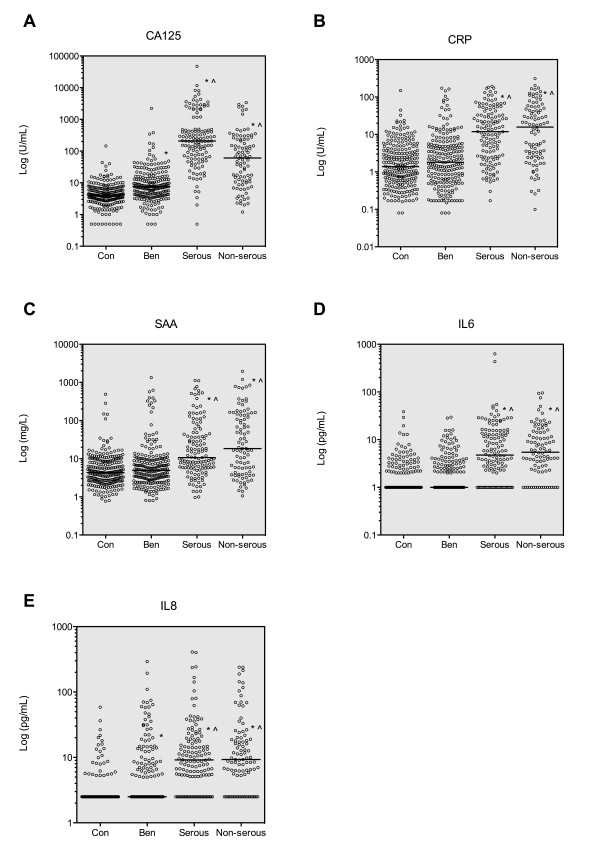
**Comparison of individual biomarker concentrations in plasma of patients with serous versus non-serous malignant ovarian cancer**. A total of 244 normal controls (Con), 223 patients with benign gynecological conditions (Ben), 130 cases of malignant serous and 92 cases of non-serous ovarian cancer were analysed. Open circles indicate the individual values of each patient measurement shown on a log scale and horizontal lines show median concentration of each group. *: *p *< 0.05, compared with Con group; ^: *p *< 0.05 compared with Ben group (Kruskal-Wallis test followed by Dunn's multiple comparison test)

The median plasma concentration of all biomarkers tested was significantly elevated (*p *< 0.05) in patients diagnosed with malignant serous ovarian carcinoma as well as ovarian carcinomas of non-serous histotypes compared with either normal controls or patients with benign conditions (Figure 2). Only circulating concentrations of CA125 were significantly different (*p *< 0.05) between patients with serous ovarian cancer compared with patients with non-serous tumours, with median CA125 concentrations of serous patients being approximately three-times higher than those in non-serous patients (Figure 2). No significant difference was found in concentrations of CRP, SAA, IL6 or IL8 between serous and non-serous ovarian cancer patients (Figure 2), suggesting that unlike CA125, these biomarkers could more effectively discriminate non-serous ovarian cancer cases from control and/or benign patients.

Further analysis demonstrated that each of the biomarkers tested could significantly discriminate between normal controls and either early (Stages I-II) or late (Stages III-IV) stage ovarian cancer patients (Table 2). Similarly, all plasma biomarkers measured were significantly higher in early stage ovarian cancer patients compared with those with benign conditions (Table 2). Only IL6 and IL8 demonstrated a significant elevation in plasma concentration between borderline tumours and early stage (Stage I-II).

### Multivariate modelling and comparisons of diagnostic performance

We first compared AUC-ROC of CA125 with that of the multimarker panel modelled using only normal controls (n = 244) and malignant ovarian cancer patients (n = 222). The AUC of the IVDMIA was significantly greater than that of CA125 alone (94.9 vs 91.9, *p *= 0.007).

Comparisons of AUC of CA125 with the IVDMIA for discrimination of control/benign from borderline and malignant epithelial ovarian cancer patients are shown in Table 3. Sensitivities are reported at the defined specificities of 90% and 95%. The use of CA125 as a single biomarker delivered an AUC of 84.3% with a sensitivity of 62.5% at either a specificity of 90% or 95%. The IVDMIA delivered an AUC of 88.4% with a sensitivity of 77.5% at 90% specificity and a sensitivity of 69.5% at a defined specificity of 95%. The AUC was significantly different (*p *< 0.001) between CA125 and IVDMIA (Table 4) and the sensitivity of the IVDMIA was 15% higher at 90% specificity and 7% higher than for CA125 at 95% specificity.

**Table 3:** ROC-AUC comparison of 5-marker multianalyte test with CA125

	ROC-AUC	95% CI	SN at 90% SP	SN at 95% SP
CA125	84.3%	80.6-87.2	62.5	62.5

5-marker panel	88.4%	85.3-91.0	77.5	69.5

Comparison by ROC-AUC analysis of 5-marker multianalyte test (CA125, CRP, SAA, IL6 and IL8) with CA125 for the discrimination of control and benign from patients with all stages of malignant epithelial ovarian cancer and borderline ovarian cancer cases. Sensitivities (SN) at selected specificities (SP) of 90% and 95% are shown.

**Table 4:** Statistical comparison of diagnostic efficiency between the 5-marker multianalyte test and CA125

Statistical Comparison	Estimate	95% CI	*p *value (bootstrap)
Δ ROC AUC	4.10	1.80-6.6	< 0.001

IDI	6.60	4.70-8.60	< 0.001

NRI	8.23	6.82-9.49	< 0.001

Comparisons of diagnostic performance for discrimination of control/benign from borderline/malignant ovarian cancer groups using AUC, IDI and NRI.

**Figure 3:**
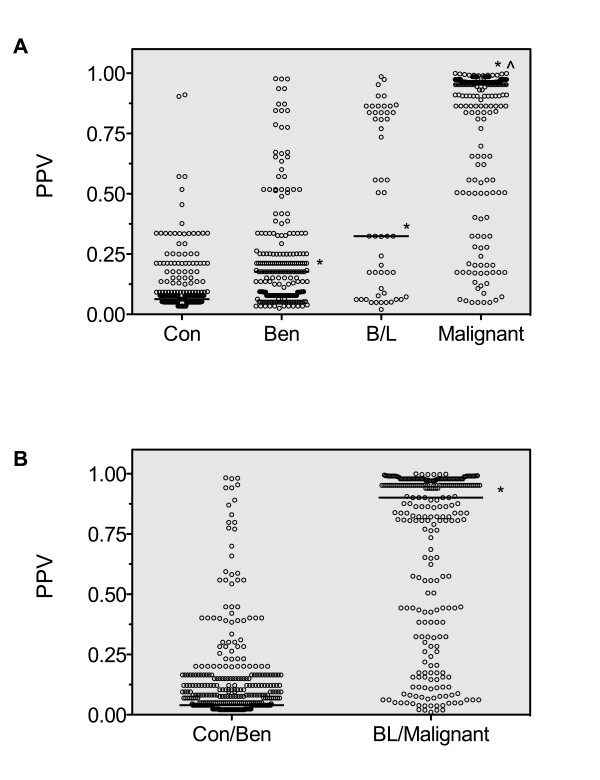
**Association between patient group and predicted posterior probability values**. **A) **Scatter plots showing the distribution of predicted posterior probability values for each patient sample across all groups and **B**), the predicted posterior probability values for each patient sample within the Control + Benign versus Borderline + Malignant groups. Horizontal bars represent median values for each group. For multi-group comparisons, *: *p *< 0.001, compared with Con group; ^: *p *< 0.001 compared with Ben group (Kruskal-Wallis test followed by Dunn's multiple comparison test). For two group comparisons, *p *< 0.0001, compared with Con + Ben group (Mann Whitney test)

The relationship between predicted posterior probability values for individual patient samples across all groups and within the combined control + benign versus borderline + malignant ovarian cancer groups is shown in Figure 3 and shows an incremental increase in posterior probability values across benign, borderline and malignant ovarian tumor patients. The algorithm derived posterior probability values for the discrimination of control/benign from borderline/malignant epithelial ovarian cancer patients was shown to be significantly different (*p *< 0.0001).

The predictive ability of the IVDMIA was further compared with that of CA125 using additional two statistical approaches (Table 4). Both the bootstrapped IDI comparison (*p *< 0.001) and NRI analysis (*p *< 0.001) demonstrated that the diagnostic performance of the IVDMIA was significantly enhanced in comparison to the use of CA125 alone for the discrimination of borderline and malignant ovarian cancer patients from the control/benign group.

Based on using a posterior probability threshold of 0.5, the multianalyte panel delivered a specificity of 92.3% and a sensitivity of 76.4%. Using this defined threshold of 0.5 for discrimination of borderline and malignant cases from the control/benign group, the multimarker algorithm correctly predicted 97.9% of normal controls, 86.1% of patients with benign lesions, 49.1% of borderline cases and 82.9% of malignant ovarian cancers of all stages. Of the ovarian malignancies, 88.5% of all serous tumours, 57.9% of all endometrioid tumours, 62.5% of all mucinous tumours, 81.2% of all clear cell tumours and 85.4% of other epithelial ovarian cancer plasma samples were correctly identified (Table 5). The IVDMIA correctly predicted 91.7% of the late stage (Stages III-IV) samples, and 69.6% of the early stage (Stages I-II) malignant ovarian cancer samples (Table 5).

**Table 5:** Proportion of samples correctly classified by the multimarker algorithm

						Correct/Total (%)
**Normal**						239/244 (97.9)

**Benign**						192/223 (86.1)

**Borderline**						26/53 (49.1)

**Malignant**						184/222 (82.9)

**Malignant EOC by Stage**						

	**I**	**II**	**III**	**IV**	**UNK**	

Ser	10/14	11/14	74/80	11/12	9/10	115/130
	(71.4)	(78.6)	(92.5)	(91.7)	(90.0)	(88.5)

Endo	4/7	1/6	4/4		2/2	11/19
	(57.1)	(16.7)	(100)		(100)	(57.9)

Muc	6/8		1/3		3/5	10/16
	(75.0)		(33.3)		(60.0)	(62.5)

CC	5/8	1/1	7/7			13/16
	(62.5)	(100)	(100)			(81.2)

Other	4/5	6/6	11/12	2/2	12/16	35/41
	(80.0)	(100)	(91.7)	(100)	(75.0)	(85.4)

Classification of samples at a threshold of 0.5 using posterior probability values derived from an algorithm based on modelling of Control + Benign versus Borderline + Malignant ovarian cancer patients.

## Discussion

The primary aims of this study were to: (i) further validate the efficacy of an IVDMIA to correctly classify ovarian cancer in symptomatic women; and (ii) to establish the utility of three different methods for assessing incremental diagnostic performance. The performance of the IDVMIA was compared with CA125 alone using AUC, IDI and NRI. An independent cohort of 742 patient samples that were derived from multiple collection sites that included a substantial proportion of women with benign gynecological conditions and borderline ovarian cancer was used to establish these aims. The diagnostic performance of the IVDMIA was shown to be superior to that of CA125 alone as demonstrated by comparison of AUC and by two new measures of performance, IDI and NRI that offer incremental information over AUC.

Each of the five biomarkers tested showed significant elevation in malignant epithelial ovarian cancer patient plasma compared with either normal control subjects or subjects with confirmed benign gynecological lesions, suggesting that each marker individually showed some ability to discriminate malignant from non-malignant conditions. Furthermore, only two of the five markers, CA125 and IL8, displayed elevated concentrations in plasma of benign patients compared with normal controls indicating that CRP, SAA and IL6 should be most effective in classifying benign lesions as non-malignant. While all of the five biomarkers examined were significantly elevated in plasma samples taken from patients diagnosed with either epithelial ovarian malignancies of serous and non-serous histotypes, only CA125 concentrations were significantly lower in the non-serous group, consistent with previous studies [6]. The differential expression of CA125 between serous and non-serous ovarian cancer histotypes suggests that additional biomarkers such as CRP, SAA, IL6 and IL8 may complement the diagnostic efficacy of CA125, particularly for non-serous histotypes.

Consistent with our previous study [8], we confirm that if the multimarker model was constructed using only the control and malignant ovarian cancer groups, the IVDMIA delivered a significant diagnostic advantage over the use of CA125 alone, indicating consistent performance of the panel in an independent sample set. The preferred multimarker model was then constructed using the broader combination of normal control and benign samples versus borderline and malignant ovarian cancer samples. When biomarker data was combined into a multivariate classification model to generate cross-validated posterior probability values to generate a ROC curve as previously described [8], the resulting AUC for the IVDMIA was shown to be significantly greater than that observed for CA125 alone for the discrimination of control/benign samples from borderline/malignant patients. Comparison of sensitivity of the multimarker panel with CA125 at a fixed specificity of 95% demonstrated enhanced performance of the multivariate index (69.5% vs 62.5%).

The multianalyte test delivered posterior probability values across the subject groups that displayed an incremental increase from benign to borderline to malignant ovarian cancer patients. Although the borderline group were the most difficult to accurately predict, combining borderline and malignant ovarian cancer patients resulted in a highly significant difference in posterior probabilities compared with the control/benign group. Using a posterior probability threshold of 0.5, the IVDMIA delivered a specificity of 92.3% and a sensitivity of 76.4%. The inclusion of patients with benign lesions and borderline tumours into the present sample cohort resulted in slightly lower overall AUC and sensitivity and specificity of both the multimarker panel and CA125 than observed in our previous biomarker trial, however, the statistically significant improvement in AUC and sensitivity and specificity over CA125 in this broader independent study cohort was maintained.

Although the AUC has become the most widely used measure of comparing performance of models for binary outcomes, it has become apparent that with a reasonably efficient marker or model, relatively small changes in AUC may not adequately describe the true clinical incremental contribution of adding new markers or of an alternative model [15]. Such observations have led to the development of new indices of classification improvement, the IDI that is based on the integral of sensitivity and specificity over all possible thresholds and can be used to quantify the increase in separation of case and controls and the NRI that is based on reclassification tables that quantify the correct movement in categories [15,16]. It has been suggested that these new measures offer incremental information over the AUC and should be considered in addition to AUC when assessing the differential performance of new models [15,16]. In order to further validate the overall diagnostic performance of the multimarker panel compared with CA125 for the discrimination of borderline and malignant ovarian cancer patients from control/benign patients, cross validated probabilities from the multimarker model were compared with CA125 by IDI and NRI. Using these alternative approaches to assess improvement in diagnostic performance also demonstrated the significant advantage (*p *< 0.001) of the IVDMIA over CA125. In summary, this study confirms, using three different statistical methodologies that the five-marker multianalyte panel provides significantly better diagnostic performance than CA125 for the discrimination of borderline and malignant ovarian cancer plasma samples from control and benign subjects.

A variety of approaches have been proposed for designing and testing multianalyte panels as aids for the diagnosis of ovarian cancer. Several multimarker panels have been shown to have considerable predictive advantage over the use of CA125 alone in different patient cohorts and settings as well as in different study designs that include retrospective, longitudinal and prospective studies [7,9-11,13,23]. While some marker panels are aimed at discriminating benign from malignant adnexal masses prior to surgery [10,11,14,24], others are being developed as panels for the early detection of ovarian cancer that may ultimately serve as part of a multi-step screening process [23]. In a prospective study of women undergoing surgery for adnexal mass, Moore *et al. *reported that the combination of CA125 and HE4 delivered significantly better discrimination of benign disease versus ovarian cancer than did CA125 alone, with a cross-validated sensitivity of 76.4% at 95% specificity [10]. A similar study, using this dual biomarker panel and separate algorithms to assess the risk of endothelial ovarian cancer in premenopausal and postmenopausal women with pelvic mass demonstrated sensitivities and specificities of 92.3% and 74.7% versus 76.5% and 74.8% in the postmenopausal and premenopausal groups respectively [25]. A more recent study assessed the clinical utility of replacing CA125 with a five-marker multianalyte test in the American College of Obstetricians and Gynecologists referral guidelines for women with pelvic mass. This study evaluated 516 women with ovarian mass and demonstrated that substituting CA125 with the multimarker test led to increased sensitivity (94% vs 77%) but decreased specificity (35% vs 68%) across all patients [14].

CA125 is still the most widely used biomarker test that shows clinical utility in the monitoring of ovarian malignancy as well as the preoperative diagnosis of suspected ovarian cancer. The present biomarker panel described here is aimed at providing an alternative to CA125 that will deliver higher diagnostic efficiency that can be used as an aid in the early preoperative diagnostic process. It has been recently suggested that a limitation of several studies examining the efficacy of biomarker panels for the early prediction of ovarian cancer is that samples are sourced predominantly from symptomatic rather than asymptomatic women [26]. Furthermore, this study suggested that several previously published multimarker panels that displayed apparently better performance than CA125 failed to do so in a study using prediagnostic samples [26]. While this is a particularly important point of consideration if the intended use of the biomarker panel is for screening of pre-symptomatic women, it is less critical if the intended use is as a diagnostic marker panel to aid assessment of symptomatic patients, as is the case with the current study.

The biomarkers measured in this study represent proteins that are known to be expressed and released from malignant ovarian epithelium as well as proteins that may be induced and released from other sites as part of an acute and/or ongoing inflammatory response or response to injury. It is well established that the CA125 epitope is contained in MUC16, a transmembrane glycoprotein that is expressed in endothelial ovarian cancer cells and subsequently shed into the circulation, thereby providing a measurable blood biomarker [27]. The acute phase protein CRP is produced predominantly by hepatocytes and its elevated concentration in the serum of ovarian cancer patients has been shown to be independently associated with FIGO stage and overall 5-year survival [28]. Circulating blood concentrations and expression of SAA, IL6 and IL8 have been shown to be correlated with ovarian tumour stage and also with patient survival [29-32]. While the source of circulating SAA, IL6 and IL8 in ovarian cancer patients is not clear, it has been demonstrated that these proteins are expressed and secreted from ovarian cancer cells [30,32,33]. Furthermore, there is evidence that IL6 and IL8 in particular can exert biological actions that influence ovarian cancer cell growth and migration [30,34,35]. Taken together, there is substantial evidence to suggest that although several of these biomarkers are traditionally considered to be systemic acute phase or inflammatory markers, expression of most of these proteins can occur locally in the malignant ovarian epithelium where they may have biological actions on the growth and development of these tumours.

## Conclusions

The study reported here is part of a larger multi-site, multi-national phase 2 biomarker evaluation and serves to validate the enhanced performance of a multianalyte panel over that of CA125 in an independent sample set comprised of borderline and malignant epithelial ovarian cancer patients and a control cohort made up of both normal women as well as women diagnosed with benign gynecological lesions. We have established a statistically significant increase in the performance of the multimarker test using the traditional and well established comparison of AUC as well as using two new measures of performance of predictive models, IDI and NRI. The current data demonstrate that the biomarker panel has utility as an improved diagnostic aid for assessing the likelihood of ovarian cancer in clinically presenting symptomatic women.

## Competing interests

This study was funded as part of the research and development activities of Healthlinx Ltd. DJA, LR, KK and KB are employees of Healthlinx Ltd and GER is non-executive chairman of Healthlinx Ltd. DJA is an inventor on patent applications that are related to the current study.

## Authors' contributions

DJA was responsible for final study design, collation of all clinical data, preparation of blinded sample lists for assay, statistical analysis, data interpretation and writing of the manuscript. GER was responsible for overall study conception, obtaining human ethics approvals and contributed to data interpretation and critical review of the manuscript. LR, KK and KB each contributed to organising clinical sample logistics, methods for sample handling, assay of all samples and collation of all raw data. Only the authors were responsible for study design, analysis and interpretation of data and writing and submission of the manuscript for publication. All authors read and approved the final manuscript.
